# Supplementary material for: Content-rich biological network constructed by mining PubMed abstracts
Source: BMC Bioinformatics. 2004 Oct 8;5:147. doi: 10.1186/1471-2105-5-147 (PMC528731; doi:10.1186/1471-2105-5-147)
Supplement: Additional File 2 — The original results of the above study (non-essential files are deleted to keep the file size under the limit set by BMC bioinformatics). [file 1471-2105-5-147-S2.bz2 › chilibotAdditionalFile2/dip10/624ID8345529E11011/html/STXA_STXB.html]

 


 **STXA** and **STXB** 
  
Found 7 abstracts in PubMed, retrieved 7.  
 

 What does Google say? 
 PDF only 
| .edu only 

---

**Interactive relationship** (e.g. stimulation, inhibition, etc)

**Neutral relationship**- The toxins are bipartite molecules composed of a single A chain  **StxA**  noncovalently associated with a pentamer of receptor binding B subunits  **StxB** .  Ref: 9649321 Biochemistry, 1998

**Non-interactive relationship** (e.g. studied together, co-existance, homology, etc.)

- The 1A 5B subunit stoichiometry of the holotoxin suggests that the  **stxA**  and  **stxB**  genes are differentially regulated.  Ref: 7678590 J Bacteriol, 1993
- Shiga toxins Stxs, encoded by the  **stxA**  and  **stxB**  genes, are important contributors to the virulence of Escherichia coli O157 H7 and other Stx producing E. coli STEC strains.  Ref: 11222608 J Bacteriol, 2001
- The  **stxA**  and  **stxB**  genes in STEC strains are located on the genomes of resident prophages of the lambda family immediately downstream of the phage late promoters p R.  Ref: 11222608 J Bacteriol, 2001
- As in the other Stx phages, a regulatory region involved in Q dependent expression is found upstream of  **stxA**  and  **stxB**  stx gene in phage 7888.  Ref: 11705937 Infect Immun, 2001
